# Hospital Meetings, &c.

**Published:** 1900-09-15

**Authors:** 


### HOSPITAL MEETINGS, &C.

### LONDON HOSPITAL.

The customary quarterly Court of Governors of the London Hospital was held on the 5th inst., Mr. John Hampton Hale presiding.

The report presented by the House Committee on the work of the past three months stated that the extensive alterations which have been in progress now for some time had naturally caused certain difficulties of administration, but the inconvenience felt by all engaged in the work of the hospital had been cheerfully accepted as inevitable, and in no way allowed to interfere with the comfort of the patients. Describing the progress made in the alterations the committee stated that the memorial raised to the memory of Sir Andrew Clarke, and which is to be devoted to the study of pathology, is now ready for the internal fittings, and it is hoped will be in use before the end of the present year. The isolation block to the east of St. Philip's Church is well advanced, and should be ready for the reception of patients early in next year. The greater portion of the front block of the hospital is in the builders' hands. The walls of the extension of the receiving room are now some feet above ground. Most of the necessary pulling down has been done, and preparations are being made to build the new operating theatres. The temporary operation theatres are now in use, and have been found in every way satisfactory. In the nursing home the kitchen accommodation has been found insufficient, and it has been found necessary to build an extension out from the basement. The committee expressed the hope that they would be able shortly to announce that the building of the new out-patient department had been begun. Certain difficulties with regard to the making of a tunnel under Oxford Street to connect the out-patient department with the hospital had arisen with the Whitechapel Board of Works, but the committee hoped to be able to make an arrangement satisfactory to both parties. The accommodation of the nurses had been recently increased by the fitting up and furnishing of two of the John Bakers Almshouses to the south of the hospital and by taking a further house, No. 60, Turner Street. A complete system of telephones had been established throughout the hospital, and this can without difficulty be extended to all parts of the new building as completed. Such satisfactory results had been obtained from the use of Professor Finsen's light apparatus for the treatment of lupus, and the applications from all parts had been so numerous, that it had been decided to put up two new instruments in addition to the one presented by the Princess of Wales. The electric light which had been installed in the temporary operating theatre, in the "Victor" operating theatre, and in the lupus department was giving satisfaction. A new cricket and sports ground had been purchased at Highams Park, the members of the staff guaranteeing interest at the rate of 4 per cent. on the outlay. On the staff there had been no changes, but it had at length been found necessary to appoint a fifth assistant surgeon. At a special court of governors held on July 30th, Mr. Harold Leslie Barnard, F.R.C.S., was elected to the post. On the recommendation of the medical council the house committee had agreed to recommend the appointment of a demonstrator in pathological histology, who would be able to direct the work in connection with the new pathological department. The Princess of Wales had selected twenty nurses from the nursing staff of the hospital for service in South Africa. On July 19th the extension of the students' club room was formally opened by Mr. Treves. Five additional beds at the Parkwood Convalescent Home had been allotted for patients in the hospital. Mrs. Gladstone's Convalescent Home, which had been closed for some time, was shortly to be opened at Mitcham, Surrey. During the quarter a further donation of £1,000 from the Grocers Company had been received, and an anonymous donation of £250 through the chapel offertory, as well as a donation of £500 to endow a cot in memory of Mr. Enthoven, a late student of the hospital. Several other donations and legacies were reported. The in-patients for the quarter numbered 3,224, of whom 334 died. There were 14,130 out-patients, and 35,851 dental and casualty cases.

In moving the adoption of the report, the Chairman said the work had been going on in a satisfactory manner; the pressure during the past two months had not been so great as that of the previous five or six. He appealed to those members of the Whitechapel Board of Works present at the meeting to use their influence with the Board to induce it to withdraw the conditions imposed on them in relation to the tunnel under the street, which were so onerous that they had put a stop to the work being carried on. The installation of the telephone, connecting eighty different points in the building, had been carried out without expense to the hospital, the cost being defrayed by a great friend of several members of the committee. The hospital was quite prepared to receive and deal with cases of plague should they be called upon to do so. At the time of the last cholera visitation they made special preparations, but happily they were not required to be brought into use. He hoped it would be so now.

The report was adopted, and some formal business having been attended to the meeting terminated.

